# RNA-seq of serial kidney biopsies obtained during progression of chronic kidney disease from dogs with X-linked hereditary nephropathy

**DOI:** 10.1038/s41598-017-16603-y

**Published:** 2017-12-01

**Authors:** Candice P. Chu, Jessica A. Hokamp, Rachel E. Cianciolo, Alan R. Dabney, Candice Brinkmeyer-Langford, George E. Lees, Mary B. Nabity

**Affiliations:** 1 Department of Veterinary Pathobiology, College of Veterinary Medicine and Biomedical Sciences, Texas A&M University, College Station, TX USA; 2 Department of Veterinary Biosciences, College of Veterinary Medicine, The Ohio State University, Columbus, OH USA; 3 Department of Statistics, College of Science, Texas A&M University, College Station, TX USA; 4 Department of Veterinary Integrative Biomedical Sciences, College of Veterinary Medicine and Biomedical Sciences, Texas A&M University, College Station, TX USA; 5 Department of Small Animal Clinical Sciences, College of Veterinary Medicine and Biomedical Sciences, Texas A&M University, College Station, TX USA

## Abstract

Dogs with X-linked hereditary nephropathy (XLHN) have a glomerular basement membrane defect that leads to progressive juvenile-onset renal failure. Their disease is analogous to Alport syndrome in humans, and they also serve as a good model of progressive chronic kidney disease (CKD). However, the gene expression profile that affects progression in this disease has only been partially characterized. To help fill this gap, we used RNA sequencing to identify differentially expressed genes (DEGs), over-represented pathways, and upstream regulators that contribute to kidney disease progression. Total RNA from kidney biopsies was isolated at 3 clinical time points from 3 males with rapidly-progressing CKD, 3 males with slowly-progressing CKD, and 2 age-matched controls. We identified 70 DEGs by comparing rapid and slow groups at specific time points. Based on time course analysis, 1,947 DEGs were identified over the 3 time points revealing upregulation of inflammatory pathways: integrin signaling, T cell activation, and chemokine and cytokine signaling pathways. T cell infiltration was verified by immunohistochemistry. TGF-β1 was identified as the primary upstream regulator. These results provide new insights into the underlying molecular mechanisms of disease progression in XLHN, and the identified DEGs can be potential biomarkers and therapeutic targets translatable to all CKDs.

## Introduction

X-linked hereditary nephropathy (XLHN) in dogs leads to chronic kidney disease (CKD) because of a defect in type IV collagen in the glomerular basement membrane (GBM). In the XLHN dogs in this study, a naturally-occurring, 10-base-pair deletion in the *COL4A5* gene located on the X chromosome results in the inability to synthesize complete α5 chains^1^. This alteration in the type IV collagen network compromises the structure and function of the GBM in both affected (hemizygous) males and, to a lesser extent, carrier (heterozygous) female dogs^2^.

XLHN in dogs is analogous to Alport syndrome (AS) in humans, as approximately 85% of people with AS have an X-linked mutation in *COL4A5* ^3^. AS is characterized by juvenile-onset CKD, ocular abnormalities, and hearing loss in affected males^4^. Thus far, only the renal abnormalities have been detected in XLHN dogs^2^. In dogs with XLHN, juvenile-onset CKD manifests as persistent proteinuria of glomerular origin as early as 3–6 months of age, followed by decreasing glomerular filtration rate and worsening azotemia, typically leading to end-stage renal failure before 1 year of age^2,5^.

Although XLHN has been studied as an example of canine CKD caused by glomerular disease and as an animal model of human AS^2^, the gene expression profile that affects progression has only been partially characterized. Furthermore, dogs with the same mutation causing XLHN display substantial variation in the rate of disease progression such that some dogs reach end-stage disease by 6 months of age and others at 12 months of age or later. Although varied times of onset and rates of progression are common among different types of mutations in people with AS^6^, disease progression may also vary among members of an AS family with an identical mutation^7^, as seen in dogs^8^.

While several studies have characterized gene expression in humans with CKD and in animal Alport models using microarrays^9,10^ or PCR^8,9,11,12^, studies that have incorporated high-throughput RNA sequencing (RNA-seq) with the objective of identifying differentially expressed genes (DEGs) and upstream regulators are lacking. Compared with traditional approaches in gene expression analysis, RNA-seq provides unprecedented flexibility in the discovery of DEGs^13^ while preserving accuracy and strong correlation with PCR^14–18^, even considering fold change levels^19^.

The objective of this study was to compare the gene expression between dogs with rapid versus slow disease progression phenotypes at 3 stages of the disease. We conducted Gene Ontology (GO) and pathway analyses to characterize DEGs among sample groups at specific time points. Since all CKDs share common pathways that lead to end-stage kidney disease^20^, the results help elucidate the molecular basis of CKD progression and thus may benefit canine patients and indicate potential therapeutic targets for AS patients.

## Results

### Histopathological evaluation of kidney biopsies

**Figure 1:**
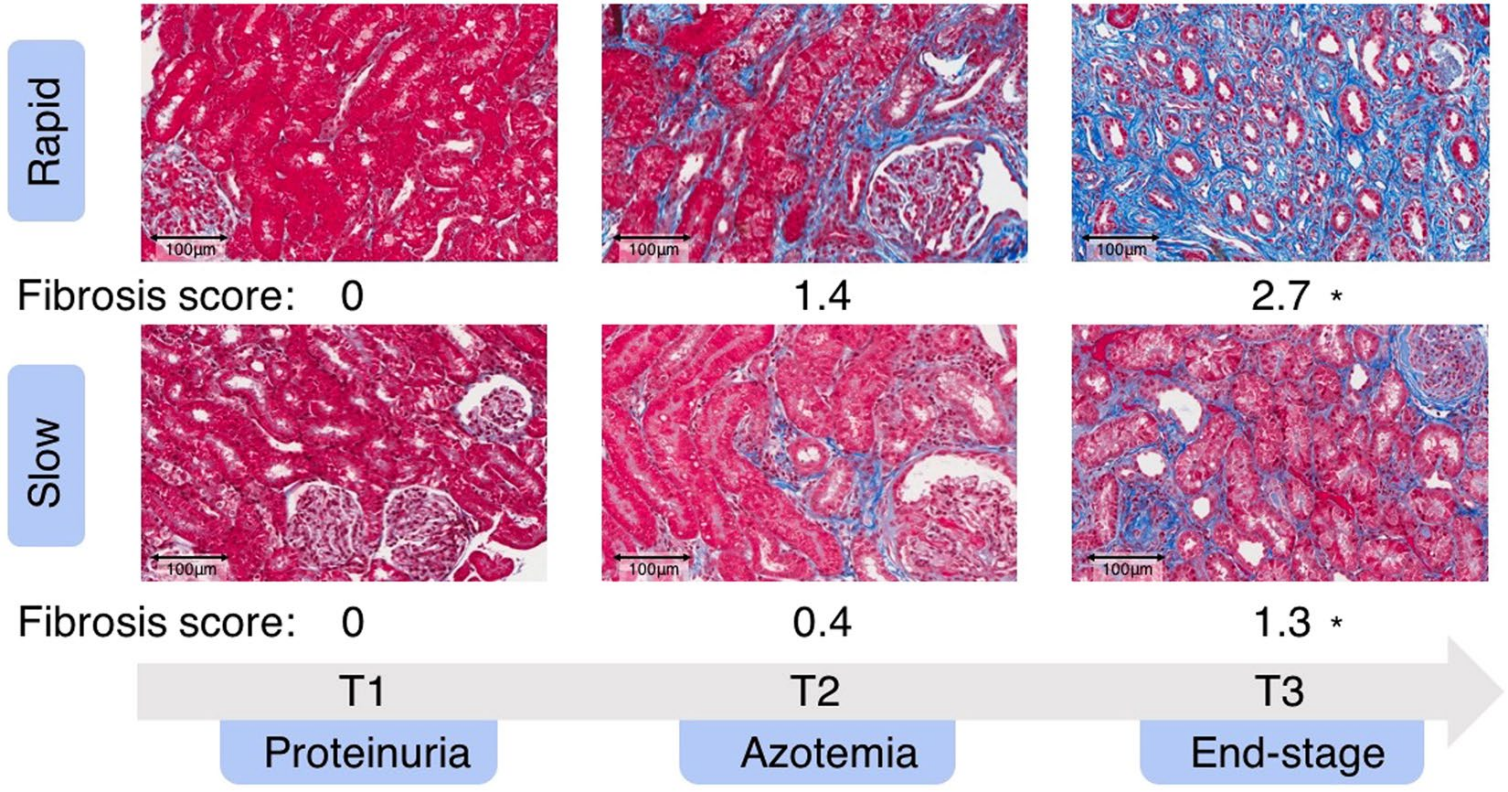
Kidney biopsies from representative dogs and mean interstitial fibrosis scores (score range: 0 [normal] to 3 [severe]) for each group of affected dogs at 3 time points (T1, T2, and T3). Fibrosis scores were based on evaluation of multiple 20x fields. *Statistical significant. Scale bar: 100 μm. (Trichrome-stained).

Figure 1 presents representative cortical fields of the kidney biopsies and the mean interstitial fibrosis scores comparing the rapid versus slow groups. Clinical time points in affected dogs were defined as: T1 - onset of proteinuria (the earliest time point that clinical disease can be detected); T2 - onset of azotemia (serum creatinine ≥ 1.2 mg/dL); and T3 - end-stage disease (serum creatinine ≥ 5 mg/dL). At both T2 and T3, mean fibrosis and chronic inflammation scores were significantly higher in diseased dogs than in controls (Supplementary Tables S1 and S2). The degree of fibrosis in the rapid group was more severe at T2 and T3 than that in the slow group, despite the two groups being clinically indistinguishable; however, statistical significance between the scores was reached only at T3 (Supplementary Table S1 and Fig. S1). No statistically significant difference was observed for the chronic inflammation score between the rapid and slow groups (Supplementary Table S2 and Fig. S2).

### RNA-sequencing (RNA-seq) of dog transcriptomes

The average RNA yield from the 24 kidney biopsies was 86.2 ng/μL, and the average RNA integrity number (RIN) was 3.4 (Supplementary Table S3). Because of the variable quality of RNA, the proper library preparation kit was used to compensate for the low-input samples according to the best practice for RNA with variable qualities^21^ (see “RNA isolation and sequencing” section in Methods). After performing quality control, we obtained an average of over 30 million paired-end reads from each sample (n = 24). Overall, 91–96% of reads were mapped to the canine genome (CanFam 3.1) by HISAT2^22^. Among them, 70–78% of reads were uniquely mapped (Supplementary Table S3 and Fig. S3). Based on the union setting of HTSeq^23^, ambiguous reads that mapped to multiple genes were not included in our analysis.

### Principal component analysis (PCA) and hierarchical clustering analysis

**Figure 2:**
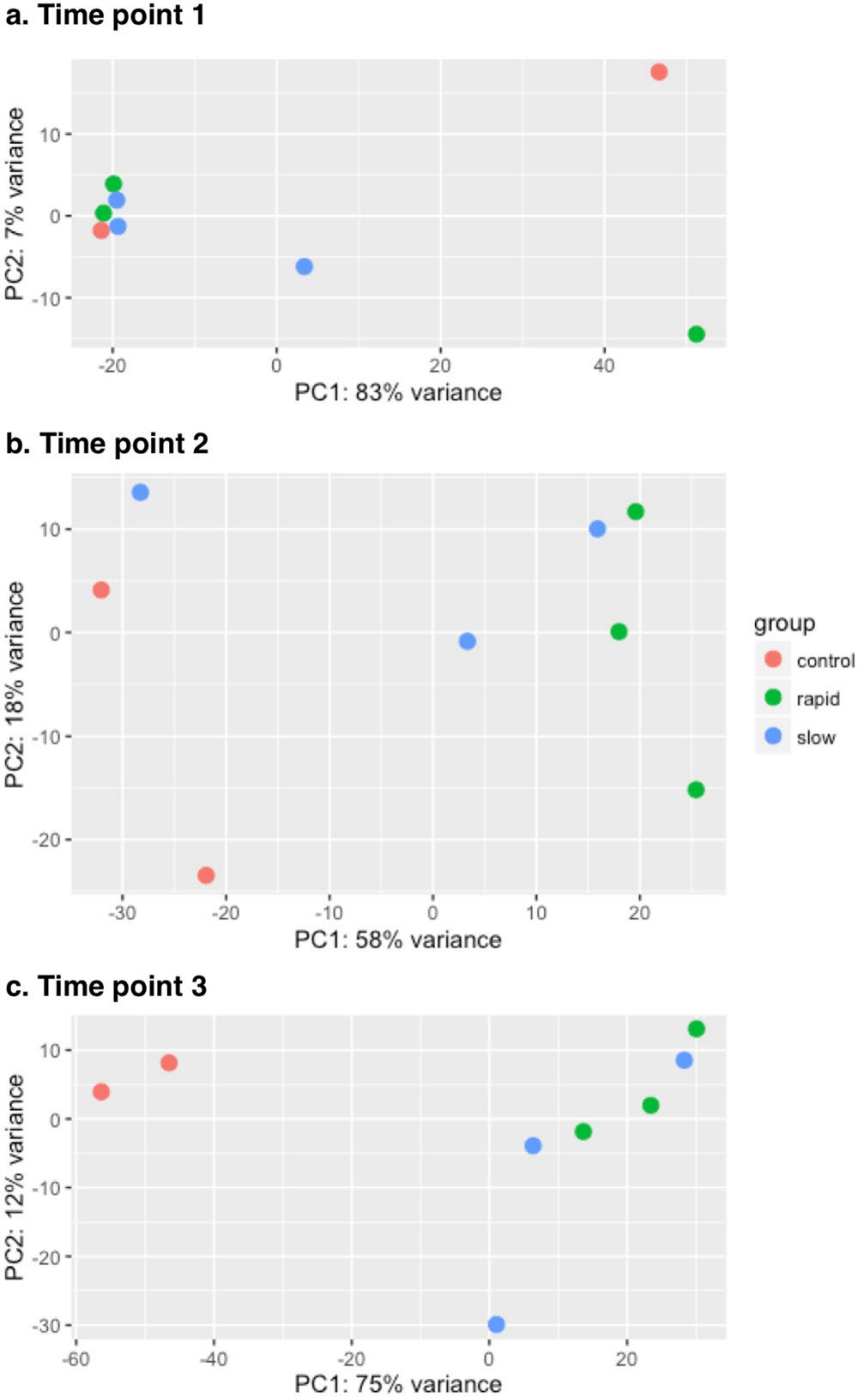
Principal component analysis (PCA) for all samples at 3 time points. Principal component 1 (PC1) and principal component 2 (PC2) were identified by variance stabilizing transformation in DESeq2 at the 3 time points. The percentage of variance indicates how much variance was explained by PC1 and PC2. (Red: control group; Green: rapid group; Blue: slow groups).

**Figure 3:**
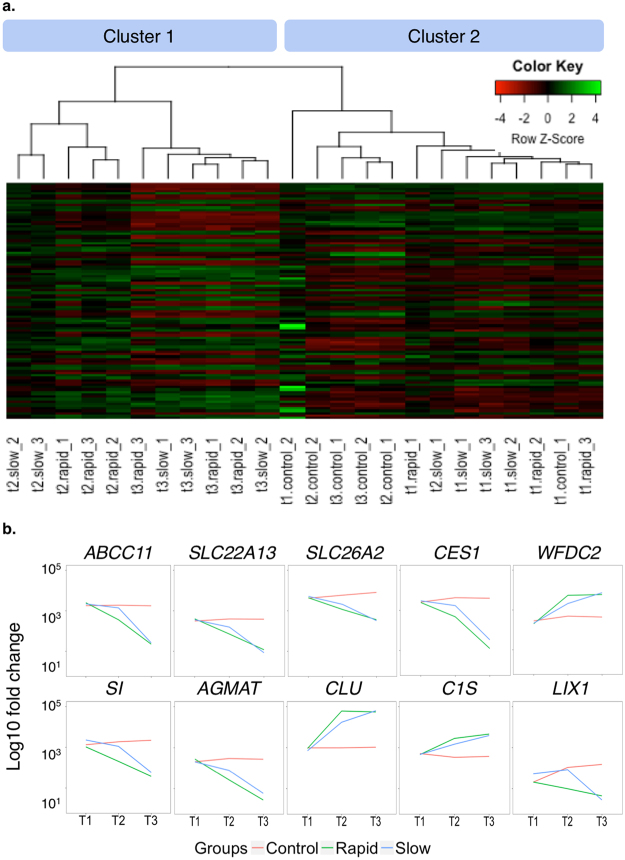
Hierarchical clustering analysis, heatmap, and gene expression. (**a**) Hierarchical clustering analysis and heatmap of the 100 genes with the smallest q-values in the time course analysis in DESeq2 (Column names: t1, t2, and t3 designate 3 clinical time points; slow, rapid, and control represent grouping; _1, _2, and _3 are individual dogs in each group). (**b**) Trends of gene expression over time for the 10 genes with the smallest q-values (from left to right, top to bottom) (Red: control group; Green: rapid group; Blue: slow group).

We performed PCA at each time point to determine whether samples in each group clustered with each other or other groups. First, we used HTSeq^23^ to count reads that uniquely aligned to one gene, and these data were then imported into DESeq2^24^ to generate PCA plots. At T1, the PCA results demonstrated that most samples clustered together, regardless of the grouping (Fig. 2). Except for one dog, rapid and slow groups became separated from controls at T2. Although the slow group tended to be closer to the controls than the rapid group, there was no clear distinction between rapid and slow groups at T2 or T3. Furthermore, PCA scree plots confirmed that principal components 1 (PC1) and 2 (PC2) accounted for 76–90% of the total variation in gene expression at each time point (Supplementary Fig. S4). To further investigate the time-dependent nature of the DEGs, we performed hierarchical clustering of the top 100 DEGs (i.e., those with the smallest q-values identified in the time course analysis in DESeq2). In agreement with the PCA plots, this analysis demonstrated clustering of almost all sample groups at T1 (Fig. 3a). At time points T2 and T3, the rapid and slow groups clustered together (Cluster 1 in Fig. 3a) and were distinctly separated from the control group for all but one T2 sample (Cluster 2 in Fig. 3a).

### Differentially expressed genes (DEGs)

In average, 20,090 genes were mapped by at least one read in each of the kidney biopsy samples (Supplementary Table S3). Overall, 1,947 DEGs with a q-value < 0.05 were detected over the 3 time points in the time course analysis of DESeq2 (Supplementary Table S4). We applied the plot counts function in DESeq2 to visualize the top 10 genes with the smallest q-values (Fig. 3b). While these genes were not differentially expressed at T1, group-specific changes were observed over time, and expression in the slow group was consistently closer to that in the control group for each gene at T2 (Fig. 3b).

**Figure 4:**
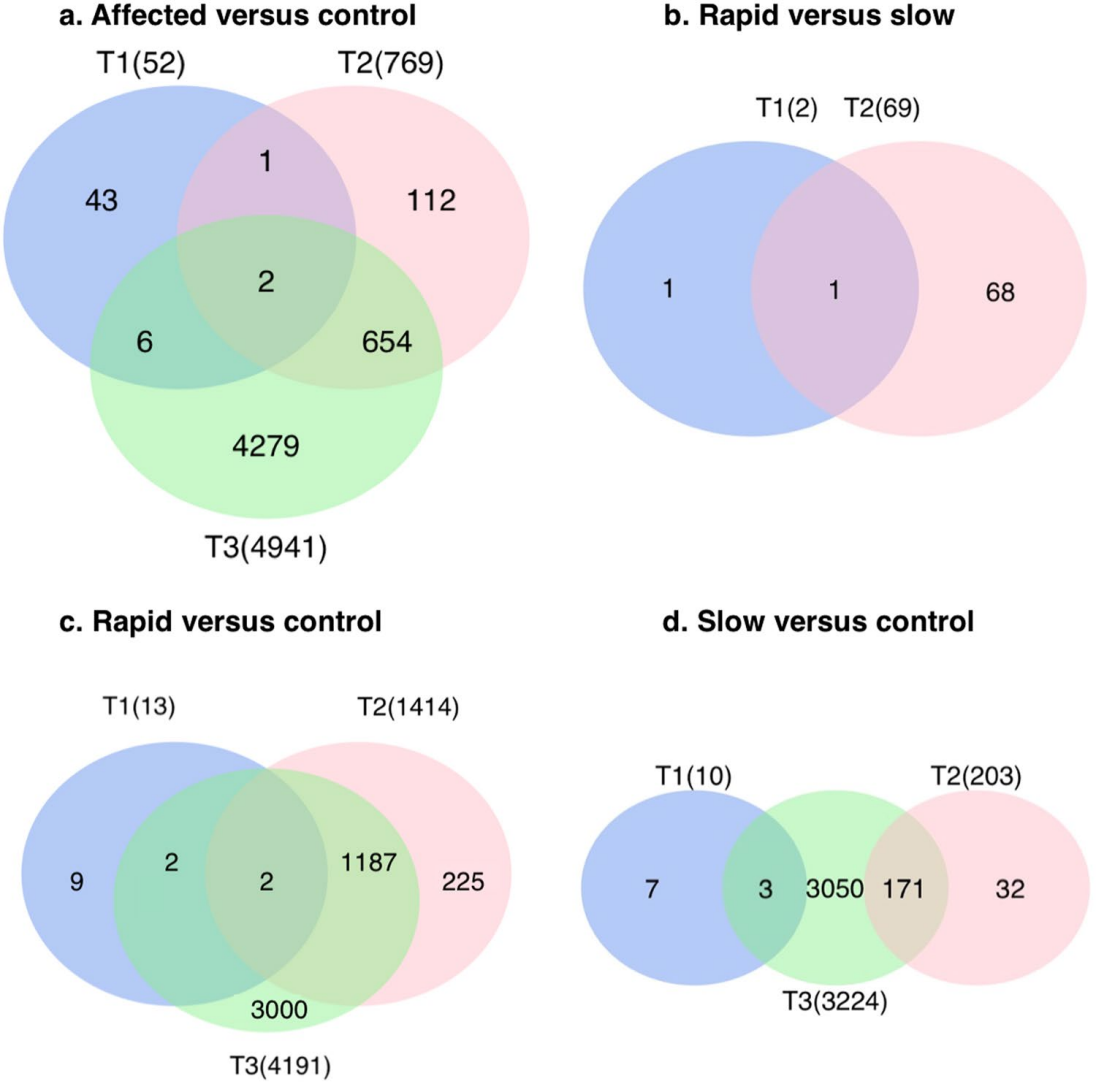
DEGs in different pairs of comparisons at the 3 time points (T1, T2, and T3). Comparing the rapid and slow groups, 70 DEGs were found. Comparing each affected group with controls, several thousand DEGs were identified, with most of the DEGs occurring at T3. For each pair, only genes with a q-value < 0.05 were considered as DEGs. The total number of DEGs found at each time point appears in parentheses.

To achieve the primary goal of this study, we identified 70 DEGs between the rapid and slow groups among all time points (q-value < 0.05) (Table 1 and Fig. 4b). In this comparison, T2 demonstrated the most DEGs, with 68 of the 70 DEGs unique to T2. Two DEGs were identified at T1: stearoyl-CoA desaturase 5 (SCD5) (fold change = −3.55, q-value = 1.1 × 10^−05^), which was also detected at T2, and thymidine kinase 1 (TK1) (fold change = 2.49, q-value = 0.02). At T3, no DEGs were identified when these 2 groups were compared.

**Table 1:** Overview of 70 significant DEGs comparing rapid and slow groups (q-value < 0.05).

Up-regulated DEGs in the rapid group			
Gene Symbol	Full Name	Fold Change	q-value
COL1A1	collagen type I alpha 1 chain	6.52	2.62E-20
COL3A1	collagen type III alpha 1 chain	5.35	1.05E-17
COL1A2	collagen type I alpha 2 chain	4.01	4.66E-13
COL5A1	collagen type V alpha 1 chain	3.8	7.21E-05
COL6A3	collagen type VI alpha 3 chain	3.27	7.08E-07
COL6A1	collagen type VI alpha 1 chain	3.26	1.82E-05
LOX	lysyl oxidase	3.13	1.42E-03
COL6A2	collagen type VI alpha 2 chain	3.07	2.34E-04
PAMR1	peptidase domain containing associated with muscle regeneration 1	2.99	5.76E-03
CDCA8	cell division cycle associated 8	2.98	2.80E-02
COL11A1	collagen type I alpha 1 chain	2.86	5.33E-03
COL15A1	collagen type XI alpha 1 chain	2.82	6.24E-03
C1QTNF6	C1q and tumor necrosis factor related protein 6	2.81	4.98E-03
FNDC1	fibronectin type III domain containing 1	2.81	3.22E-02
FN1	fibronectin 1	2.8	4.10E-02
CCDC80	coiled-coil domain containing 80	2.72	7.98E-04
MFSD7	major facilitator superfamily domain containing 7	2.7	3.22E-02
FBLN1	fibulin 1	2.69	4.10E-02
NID2	nidogen 2	2.61	1.52E-07
COL4A2	collagen type IV alpha 2 chain	2.6	1.42E-03
FAM69B	family with sequence similarity 69, member B	2.6	3.42E-02
NDN	necdin	2.59	4.39E-02
COL4A1	collagen type I alpha 1 chain	2.59	5.76E-03
HTR7	5-hydroxytryptamine (serotonin) receptor 7, adenylate cyclase-coupled	2.58	2.60E-02
PCOLCE	procollagen C-endopeptidase enhancer	2.58	5.83E-03
OLFML2B	olfactomedin like 2B	2.56	6.24E-03
TK1^a^	Thymidine kinase 1	2.49	1.98E-02
C15orf39	chromosome 15 open reading frame 39	2.47	2.76E-02
RCN3	reticulocalbin 3	2.46	6.94E-04
MFAP2	microfibrillar associated protein 2	2.45	1.42E-03
HSPG2	perlecan	2.45	1.20E-02
PRSS35	protease, serine 35	2.45	4.10E-02
MMP2	matrix metallopeptidase 2	2.43	4.48E-05
FBN1	fibrillin 1	2.37	3.57E-03
CD248	CD248 molecule	2.36	2.72E-02
FOXRED2	FAD dependent oxidoreductase domain containing 2	2.35	1.11E-02
GXYLT2	glucoside xylosyltransferase 2	2.34	3.62E-02
FSCN1	fascin actin-bundling protein 1	2.34	1.20E-02
ENSCAFG00000008741^b^	novel gene	2.29	1.18E-02
ENSCAFG00000012963^b^	novel gene	2.28	1.23E-03
BGN	biglycan	2.19	1.85E-02
FAS	Fas (TNF receptor superfamily member 6)	2.16	2.08E-02
ADAMTS2	ADAM metallopeptidase with thrombospondin type 1 motif 2	2.15	3.23E-02
PXDN	peroxidasin	2.14	4.10E-02
SPARC	secreted protein acidic and cysteine rich	2.13	4.48E-05
THBS1	thrombospondin 1	2.12	1.26E-04
KCP	kielin/chordin-like protein	2.12	4.19E-02
LRP1	LDL receptor related protein 1	2.11	7.98E-04
CERCAM	cerebral endothelial cell adhesion molecule	2.11	4.39E-02
ITGA5	integrin subunit alpha 5	2.07	7.03E-03
BMP1	bone Morphogenetic Protein 1	1.88	6.74E-05
FSTL1	follistatin Like 1	1.72	1.25E-05
PTGFRN	prostaglandin F2 Receptor Inhibitor	1.66	2.61E-05
**Down-regulated DEGs in the rapid group**			
UGT1A6	UDP glucuronosyltransferase family 1 member A6	−4.77	1.23E-03
NAT8	N-acetyltransferase 8 (putative)	−4.1	1.09E-04
R3HDML	R3H domain containing like	−4.02	1.42E-03
LIX1	limb and CNS expressed 1	−3.83	7.21E-03
PRLR	prolactin receptor	−3.73	4.64E-04
SCD5^c^	stearoyl-CoA desaturase 5	−3.55	1.07E-05
FMO2	flavin containing monooxygenase 2	−3.42	1.78E-02
OAT3/SLC22A8	solute carrier family 22 member 8	−3.37	2.45E-02
SI	sucrase-isomaltase	−3.25	1.16E-02
ENSCAFG00000003760^b^	novel gene	−2.92	3.96E-02
SLC26A4	solute carrier family 26 member 4	−2.79	7.14E-03
ENSCAFG00000000799^b^	novel gene	−2.77	3.25E-02
HEPACAM2	HEPACAM family member 2	−2.66	8.81E-03
IDO2	indoleamine 2,3-dioxygenase 2	−2.58	3.42E-02
PECR	peroxisomal trans-2-enoyl-CoA reductase	−2.57	6.24E-03
MT-ND3	mitochondrially encoded NADH dehydrogenase 3	−2.16	3.49E-03
ABCA4	retinal-specific ATP-binding cassette transporter	−1.91	1.08E-04

^a^DEG identified only at T1. ^b^Genes are displyed with ensembl IDs if gene annotations are unavailable. ^c^DEG identified at both T1 and T2.

We also compared the rapid and slow groups with the control group, both individually and combined as a single “affected” group (Fig. 4a,c, and d). In these comparisons, the number of DEGs increased with advancing disease, with the largest number of DEGs identified at T3. This phenomenon indicates that the DEGs are disease-dependent as they are more differentially expressed in the later time points (T2 and T3) than T1 (Fig. 4).

**Figure 5:**
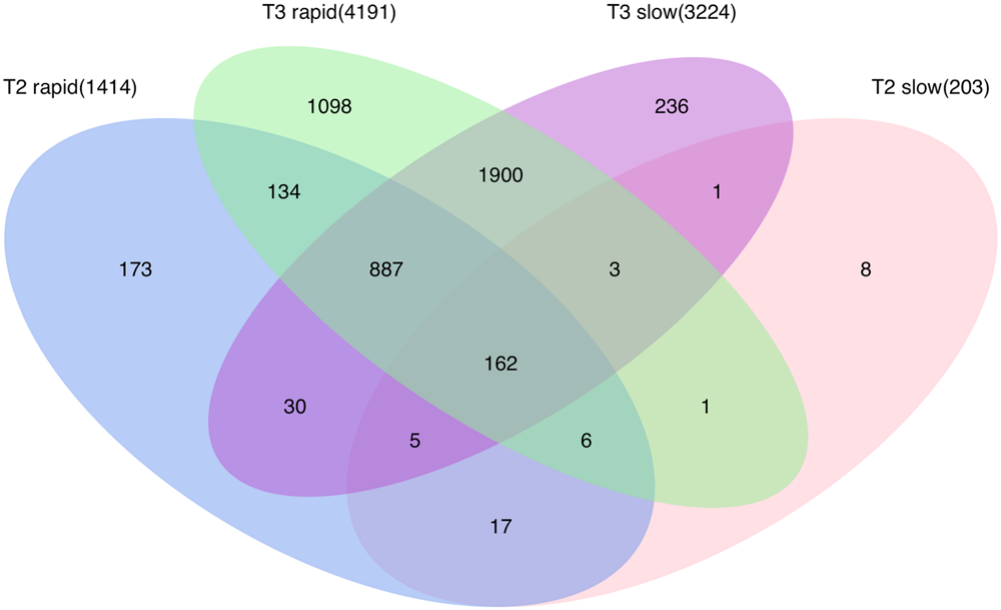
Overlapping DEGs in rapid and slow groups compared with control at T2 and T3. For each comparison, only genes with a q-value < 0.05 were considered as DEGs. The total number of DEGs found at each time point appears in parentheses.

A substantial overlap of DEGs was present when comparing rapid and slow groups with the control group at T2 and T3 (Fig. 5). The overlapping DEGs between the rapid and slow groups were more numerous at T3 (2,952 DEGs) than at T2 (190 DEGs), supporting that the two groups behave similarly at the end-stage disease, as expected based on Figs 2–4. Furthermore, the number of DEGs (1,189 DEGs) identified in both T2 and T3 in the rapid group was higher than the number of DEGs (171 DEGs) identified in both T2 and T3 in the slow group. This supports the theory that rapidly-progressing dogs express end-stage DEGs at a young age. The complete lists of DEGs from the time course analysis and all pairs of comparisons appear in Supplementary Table S4.

### Gene Ontology (GO) and pathway analysis of DEGs

**Figure 6:**
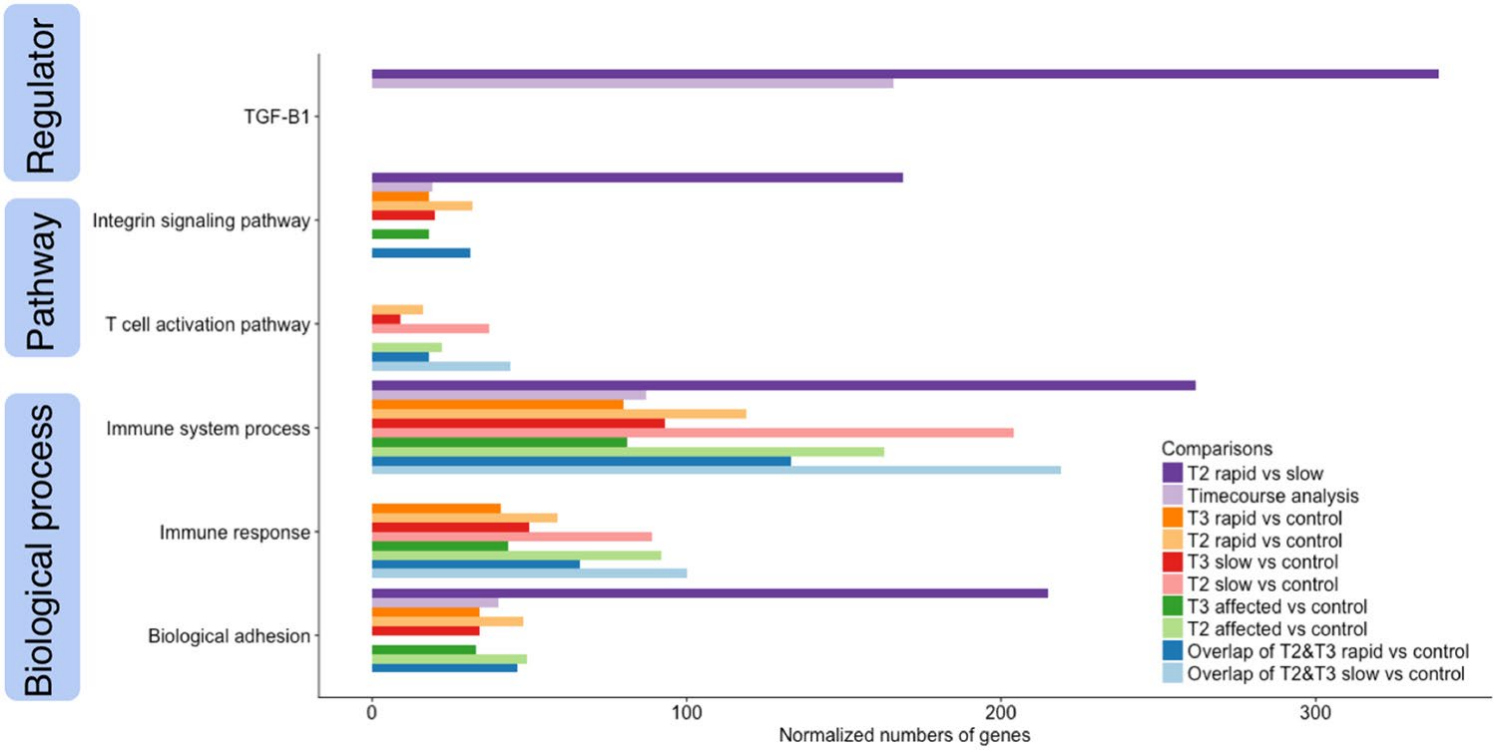
Enriched pathways and GO term analyses for 10 selected comparisons. Enriched pathways, biological processes, and regulator analyses for all DEGs are presented based on T2 and T3 comparisons as well as a time course analysis incorporating all time points for all groups. The number of genes was normalized to allow comparisons between groups within the same pathway, and comparisons were color coded in pairs, with the darker color corresponding to the later time point or the rapid group.

To characterize the GO terms, including molecular functions, biological processes, cellular components, and functional pathways of DEGs, we conducted over-representation tests for all pairs of comparisons in PANTHER version 11.1 (released on October 24, 2016) (Fig. 6 and Supplementary Table S5). We used the GO-Slim PANTHER annotation data set, which represents phylogenetically inferred annotations^25^.

Overlaps of GO terms among comparisons were commonly seen in the current study. We focused on “biological process” since it is the most characterized GO term. Within this category, the “immune system process” family was upregulated in all 10 comparisons presented in Fig. 6, and the “immune response” family was upregulated in all except for the rapid versus slow comparison at T2 and the time course analysis. Both immune-related GO terms appeared to be more upregulated at T2 than T3. At T2, the “biological adhesion” family, especially the cell-cell adhesion subfamily, was expressed in the rapid group more than in the slow group (Supplementary Table S5).

The most common pathway represented by the DEGs within the various comparisons was the “integrin signaling pathway” (Fig. 6). This was the only pathway identified in the comparison between rapid and slow groups. It was also the top upregulated pathway at T2 comparing rapid and control groups. Interestingly, analyzing the overlapping DEGs between T2 and T3, the integrin signaling pathway was identified as the top upregulated pathway within the rapid group, but not within the slow group, as compared with control.

The “T cell activation pathway" was another frequently detected pathway in our study (Fig. 6). It was upregulated in all comparisons with control at T2 and in overlapping DEGs between T2 and T3 within both the rapid and the slow groups. Another upregulated pathway included the “inflammation mediated by chemokine and cytokine signaling pathway” that was identified only when comparing all affected dogs with controls at T2. Because only limited numbers of DEGs were discovered, no enriched pathways were identified at T1 with any comparison.

To further explore the possible biological interaction between orthologous genes in the human, mouse, and rat, we performed Ingenuity Pathway Analysis (IPA) to discover the most prevalent pathways and upstream regulators within each comparison. The “hepatic fibrosis/hepatic stellate cell activation pathway” was identified as the top pathway in multiple comparisons (Supplementary Table S6). Transforming growth factor beta 1 (TGF-β1) was the most activated upstream regulator when the rapid and slow groups were compared at T2 and in the time course analysis (Supplementary Table S6).

### Immunohistochemistry (IHC) validation of inflammatory pathways

**Figure 7:**
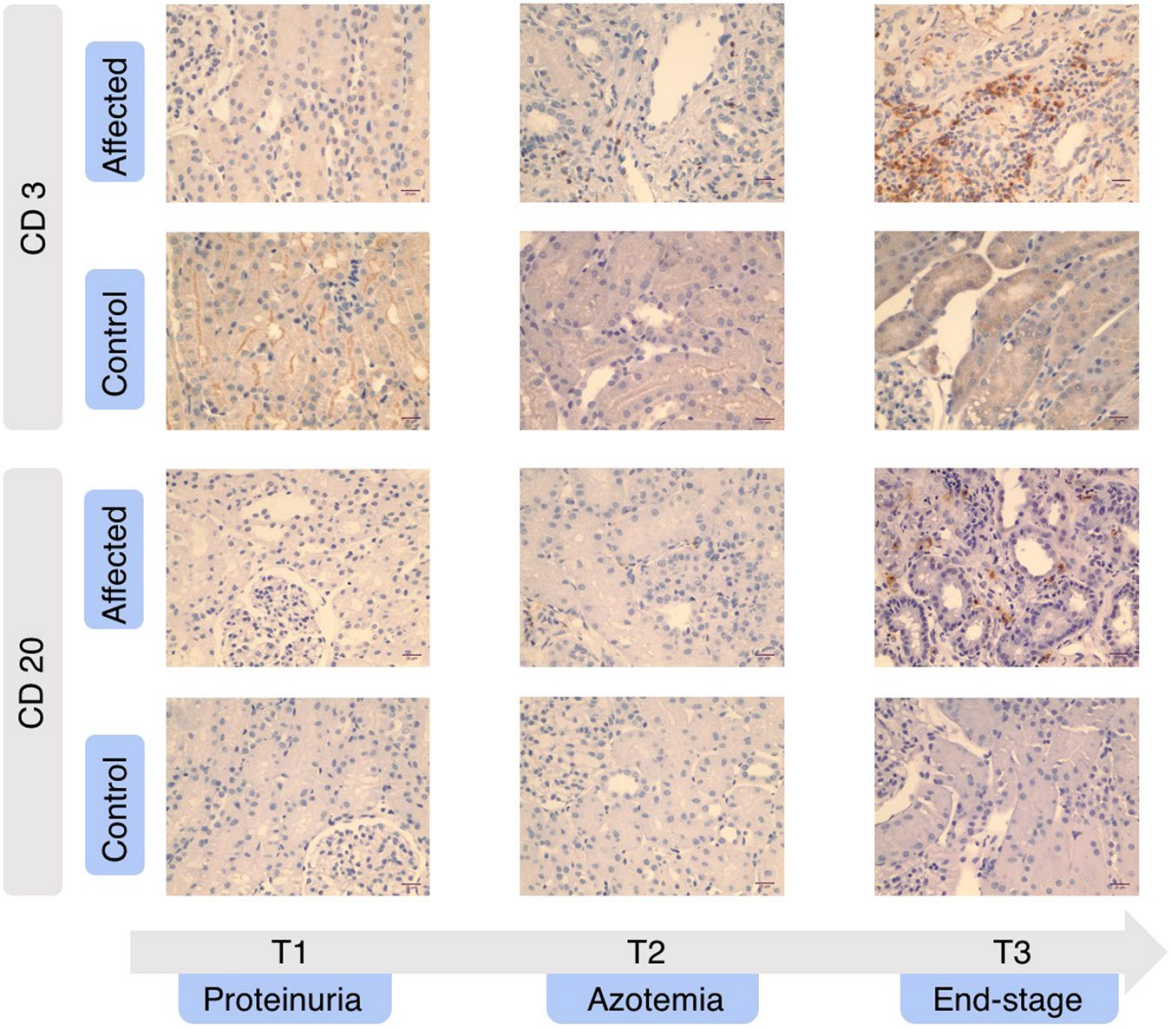
Expression of CD3 and CD20 using immunohistochemistry (IHC) in kidney biopsies from representative dogs. In affected dogs, lymphocyte infiltration present at later time points consisted mostly of CD3-positive lymphocytes with few CD20-positive lymphocytes identified. Scale bar: 20 μm.

Lastly, we aimed to validate the overexpression of inflammatory pathways in kidney biopsies. We chose to identify the predominant lymphocyte subtype present (T versus B cell) since the identified inflammatory pathways are all closely related to the presence of T lymphocytes^26^ and antibodies for CD3 (T cell) and CD20 (B cell) are validated for use in dogs^27,28^. As shown in Fig. 7, we confirmed the lymphocyte infiltration in the affected dogs to be composed mostly of T cells rather than B cells. This result correlated with the previously assigned chronic inflammation scores based on the histopathological evaluation (Supplementary Table S2 and Fig. S2) and validated our RNA-seq data.

## Discussion

Dogs with XLHN have been studied as both an example of progressive canine glomerular disease and an animal model of human AS, which has CKD as a major syndrome component^2^. The genetic cause is well characterized; however, the gene expression and molecular pathways influencing disease progression are incompletely known. In particular, the variable rate of disease progression in dogs with the same mutation and within families affected by AS is intriguing^7^. We, therefore, aimed to evaluate differential gene expression, overrepresented pathways, and upstream regulators by comparing RNA-seq data in dogs that displayed a rapid clinical progression of the disease to those with relatively slow disease progression.

In this study, we examined serial biopsies from rapid and slow groups as well as healthy age-matched littermates. To understand the biological changes during the pathogenesis of CKD, we included the earliest time point at which clinical disease could be detected in these dogs (onset of proteinuria, T1), with the onset of azotemia (T2) and the advent of end-stage disease (T3). By performing renal biopsies when animals reached specific clinical markers of disease progression, we could compare the same clinical stage in the rapid and slow groups. We believe this type of approach provides more confidence in identifying DEGs that are involved in the rate of disease progression than the traditional method of using age-driven time points, as differences detected are likely to be the driving force rather than the consequence of disease progression. Our data demonstrate the dynamic changes in gene expression at different stages of the disease. It also supports that the biological processes and pathways of fibrosis/adhesion and inflammation are the likely driving forces producing different rates of disease progression in the rapid and slow groups.

While it is known that serum creatinine correlates strongly with tubulointerstitial fibrosis^29^, one of the most intriguing findings when comparing the rapid and slow groups was the increase in fibrosis observed in the rapid group at the same clinical stage of disease (T2 and T3; Fig. 1). This corresponds with many of the 70 DEGs identified using RNA-seq between the rapid and slow groups that are implicated in fibrosis, almost all of which were identified at T2. Several of these genes have been previously described as upregulated in XLHN dogs, Alport mice, and other kidney diseases^9,11,30,31^. Among these, one of the upregulated genes, *CD248* (endosialin or tumor endothelial marker 1, *TEM1*), has been found to mediate the adhesion and migration of cells through ligand interaction with the upregulated collagen-related genes: collagen type I, collagen type IV, and fibronectin-1 (*FN1*)^32^. CD248+ stromal cells bind extracellular matrix and have been implicated in kidney^33^, liver^34^, and lung fibrosis^35^. In non-inflamed kidneys, CD248 is expressed by mesangial cells located in glomeruli. In fibrotic kidneys, CD248 is additionally expressed by myofibroblasts and stromal fibroblasts, and the increased expression is closely related to prognostic indicators, such as albuminuria and renal scarring^33^. Of note, one of the downregulated genes in the rapid group, prolactin receptor (*PRLR*), decreased in association with the extent of interstitial collagen I deposition in kidney transplant rejection^36^, suggesting that PRLR might be a protectant against renal fibrosis. In our study, the decrease in *PRLR* could be responsible for the more rapid development of fibrosis and consequential faster progression of disease in the rapid group.

Involvement of inflammatory components in the progression of CKD is another major finding of this study. Several inflammatory genes involved in fibrotic changes, such as biglycan (*BGN*), kielin/chordin-like protein (*KCP*), and matrix metallopeptidase-2 (*MMP2*) were upregulated in the rapid versus slow groups. BGN plays a role in bone growth, muscle development and regeneration, and collagen fibril assembly in multiple tissues. *BGN* is upregulated in renal fibrosis^37^, and BGN protein expression strongly correlated with chronic kidney progression in one study^30^, which may suggest its role in regulating inflammation and innate immunity. KCP expression is stimulated by renal stress, and it enhances the antifibrotic function of BMP7 to attenuate the profibrotic stimulus of TGF-β and to suppress proinflammatory cytokines^38^. In Alport mice, the administration of recombinant BMP7 reduces glomerular and interstitial fibrosis but also upregulates MMP2^39^. This upregulation of MMP2 seems contradictory, as it is associated with renal fibrosis in several animal models, including XLHN dogs^11^. However, the function of MMP2 is specific to the temporal context of fibrosis. At the prefibrotic phase, increased MMP2 induces epithelial to mesenchymal transition, tubular atrophy, and fibrosis. For established fibrosis, inducing MMP2 synthesis by BMP7 promotes proteolytic removal of accumulated extracellular matrix, which is thought to be a potential therapeutic strategy^40^. Since both KCP and MMP2 are upregulated at T2, when fibrosis is already relatively well established in the rapid group, their downstream actions are likely skewed toward anti-fibrotic effects.

Mechanisms other than fibrosis and inflammation can also play roles in the rapid progression of CKD. NAT8, which is almost exclusively expressed by tubular cells in the renal cortex^41^, is a cysteine S-conjugate N-acetyltransferase that is responsible for glutathione-mediated detoxification of nephrotoxic substances. The downregulation of *NAT8* in the rapid group suggests a more severe loss of normal renal function in the presence of similar serum creatinine concentrations compared with the slow group. Another downregulated gene, organic anion transporter 3 (*OAT3*, also known as *SLC22A8*), is decreased in kidney biopsies from human CKD patients and in a nephrectomized rat model of CKD^42,43^. Reduced protein expression of Oat3 is associated with decreased excretion of an endogenous uremic toxin; meanwhile, the accumulation of this uremic toxin further inhibits Oat3-mediated transportation, accelerating toxin accumulation in serum^42,43^. Therefore, downregulation of *OAT3* in the rapid group may result in impaired urinary excretion that is not adequately represented by serum creatinine concentration.

Only 2 DEGs, SCD5 and TK1, were differentially expressed in the rapid group as compared with the slow group at T1. SCD5 was downregulated at both T1 and T2 in the rapid compared with the slow group. And, it was downregulated in the rapid versus control comparison throughout all 3 time points. SCD5 is an isoform of stearoyl-CoA desaturase that is responsible for the formation of monounsaturated fatty acids. Although SCD5 has not previously been described in the CKD literature, it has been proposed as a novel regulator of neural cell proliferation and differentiation, likely through β-catenin-independent (non-canonical) Wnt pathways^44^. In fibrotic kidneys, the canonical Wnt pathway induces myofibroblast differentiation, and the non-canonical Wnt pathway leads to cytoskeleton rearrangement, cell adhesion, and cell movement^45^. Thymidine kinase 1 (*TK1*) is the only DEG that was exclusively upregulated at T1. Thymidine kinase is responsible for producing dTMP that is later incorporated into DNA. The cytoplasmic isoform of TK1 is cell cycle-dependent, as it substantially increases in the S phase of the cell cycle. Given that unregulated proliferation is the hallmark of neoplasia, TK1 is a valuable serum marker for breast cancer, non-Hodgkin’s lymphoma, plasmacytoma, and lung cancer^46^. Its upregulation in our study could indicate increased cell proliferation at T1 in the rapid group. However, further investigation of SCD5 and TK1 is needed to determine their roles in CKD progression.

In addition to abovementioned genes, many genes that are rarely described in CKD progression were found differentially expressed in the rapid versus slow groups at T2 (e.g., *LOX, PAMR1, CDCA8, C1QTNF6, FNDC1, CCDC80, MFSD7, FMO2, SI, SLC26A4*). The protein product of the upregulated gene lysyl oxidase (*LOX*) is an extracellular enzyme that is essential for covalent cross-linking of collagen in irreversible extracellular matrix deposition^47^. Despite reports of its upregulation in liver fibrosis^48,49^ and cardiomyopathy^50^, the upregulation of LOX in CKD has only been described in one study using a glomerulonephritis mouse model^51^. Simtuzumab, a monoclonal antibody that inhibits one of the LOX family members, lysyl oxidase homologue 2 (LOXL2), has recently been a focus of research as a possible new treatment for lung, liver, and kidney fibrosis due to a similar pathogenesis^52^. Another gene minimally described in nephrology is the downregulated sucrase-isomaltase (*SI*). SI is an α-glucosidase that commonly appears on the brush border of small intestinal enterocytes and is involved in glucose digestion. SI is also present in small amounts in non-intestinal cells such as blood leukocytes and kidney cells^53^. Little is known about the function and significance of SI in the renal tubule. However, the decreased expression of SI could indicate renal damage.

To characterize DEGs in functional groups, we performed GO terms and pathway analyses by comparing affected dog groups, both individually and collectively, with control dogs at each time point. GO terms showed that the functions of identified DEGs were associated with “biological adhesion,” “immune system processes,” and “immune response,” representing a common mechanism of disease progression in the early stages of CKD^54^. Consistent with the GO terms, the “integrin signaling pathway” was the most upregulated pathway, and the “T cell activation pathway” and the “chemokine and cytokine signaling pathway” were also upregulated in multiple comparisons. The exclusive early expression of the “integrin signaling pathway” in the rapid group and universal expression at a later time point could indicate that it is an essential pathway driving rapid progression of disease in these dogs. The “integrin signaling pathway” consists mainly of collagen and integrin genes. The integrin subunit alpha 2 gene (*ITGA2*) was increased in the rapid versus control group at both T2 and T3. The COL4A3^−/−^/ITGA2^−/−^ double knockout Alport mouse model has delayed renal fibrosis compared with COL4A3^−/−^/ITGA2^+/+^ Alport mice, which express significantly higher levels of MMP2, MMP9, MMP12, and TIMP1^55^. Upregulation of *MMP2* is consistent with our Alport dog model, suggesting that the “integrin signaling pathway” is involved in matrix accumulation.

To verify the pathway analysis results, we used IHC to identify the infiltrating lymphocyte population. The “T cell activation,” “integrin signaling,” and the “inflammation mediated by chemokine and cytokine signaling” pathways are all closely related to the presence of T lymphocytes^26^. Consistent with this, T lymphocytes were identified as the predominant inflammatory cell population present in the affected dogs, as well as in a study of canine end-stage renal disease^56^. Moreover, T cell infiltration inversely correlates with renal function at the time of renal biopsies in AS patients^57^. Overall, IHC validated the results of GO terms and pathway analyses, which showed that inflammatory pathways and corresponding biological processes are altered during the progression of CKD.

Ingenuity Pathway Analysis (IPA) allows for characterizing orthologous genes, and results identify possible mechanisms that have been validated in humans, mice, and rats. The top upregulated pathway identified in multiple comparisons was the “hepatic fibrosis/hepatic stellate cell activation pathway.” The IPA identified enriched pathways based on the over-represented DEGs, and the principle is the same as that used in the GO terms analysis via Panther. There was an extensive overlap between the DEGs we identified and the genes involved in the “hepatic fibrosis/hepatic stellate cell activation pathway” in the IPA, including collagen genes, cytokine-related genes, matrix metalloproteinases, tissue inhibitor of metalloproteinase, and the TNF receptor superfamily. Therefore, the upregulation of this pathway in our study supports common mechanisms involved in hepatic and renal fibrosis. It could also indicate contributing genes beyond those identified using known canine gene pathways.

The upstream regulator analysis of IPA identified the TGF-β group, especially TGF-β1, as the top upstream regulator, with both the highest activated z-score and the lowest p-value across multiple comparisons. Previous studies in canine^8^ and murine^58^ models of AS demonstrated the expression of TGF-β mRNA in kidney tissue. However, TGF-β was not differentially expressed in our comparisons, and IHC staining for TGF-β did not demonstrate appreciable differences in XLHN dogs compared to controls in a previous study^8^. The IPA Upstream Regulator Analysis predicts the upstream regulator of gene expression changes based on the knowledge of this regulatory cascade in the literature compiled in the Ingenuity Knowledge Base. Thus, the identified upstream regulator may not be identified as a DEG despite its importance in gene regulation.

A limiting factor of this study is that the kidney biopsies were immediately placed into RNALater to preserve RNA integrity, so microscopic evaluation could not be performed to determine whether the biopsy used for RNA isolation was representative of the cortex as a whole. Thus, one of the samples in the control group at T1 could have represented an area affected by a clinically insignificant insult. Another limiting factor is that expression data represents the mean expression by many cell types. Because the kidney has many cell types, all with different roles in CKD progression, it would be ideal to study gene expression changes in individual cells using laser-capture microdissection to further elucidate the progression of CKD. Last, extensive validation of the RNA-seq results was not performed; however, previous studies have shown RNA-seq to be a robust tool that highly correlates with qPCR results^14–19^. RNA-seq may even be more reliable than qPCR due to its higher sensitivity and lower probe bias^16^. We did perform IHC to further characterize the inflammatory population, which supported the pathways identified through the RNA-seq results.

In what appears to be the first RNA-seq study of a canine CKD model, we identified several previously described and novel genes and enriched pathways involved in the pathogenesis and development of CKD. The approach of acquiring biopsies at time points determined by the clinical stage of disease was an attempt to target causative gene expression starting at the clinical onset of proteinuria rather than secondary changes. Regardless of initial insult, CKD has common pathways that lead to end-stage kidney disease^20^. Therefore, many genes found in the current study may serve as predictive or diagnostic biomarkers for early detection of CKD in dogs and people. They may also be potential targets for drug development for this condition.

## Methods

### Animals

The dogs in this study were part of a colony with XLHN maintained at Texas A&M University^5^. XLHN is caused by a 10-base deletion in the gene encoding the α5 chain of type IV collagen. Affected males develop juvenile-onset CKD that progresses to end-stage renal disease as previously described^5^. Overall, 6 affected dogs and 2 unaffected littermates were studied. All dogs were raised according to standardized protocols, and no treatments were given to these dogs. All protocols were approved by the Texas A&M University Institutional Animal Care and Use Committee.

### Clinical phenotypes

For this study, dogs were selected to represent both extremes in the speed of disease progression in this family of dogs (rapid versus slow progression). Clinical progression was determined by serial monitoring of serum and urine biomarkers of kidney disease, which allowed us to establish specific progression time points^29^: T1 (onset of proteinuria: defined as the presence of microalbuminuria for 2 consecutive weeks (E.R.D. HealthScreen Canine Urine Test Strips, Loveland, CO, USA)); T2 (onset of azotemia: serum creatinine ≥ 1.2 mg/dL); and T3 (end-stage disease: serum creatinine ≥ 5 mg/dL). Rapidly-progressing (rapid) dogs (n = 3) reached each time point at an earlier age than slowly-progressing (slow) dogs (n = 3). On average, the rapid group reached T3 at 26.3 weeks of age (range: 26–27 weeks), while the slow group reached the last clinical time point (T3) at 49 weeks of age (range: 46–52 weeks) (Supplementary Table S7).

### Tissue collection

Kidney cortex was serially collected from each dog at the aforementioned 3 clinical time points (independent of age). Control dogs (n = 2) were biopsied to correspond with an affected littermate. All samples were collected by ultrasound-guided needle biopsy. This technique was appropriate for the current study as it is unlikely to induce changes that might be confused with those of CKD progression^59^. Samples for pathology evaluation and immunohistochemistry were placed in formalin and embedded in paraffin. Samples for RNA sequencing were immediately placed in RNAlater Stabilization Solution (Life Technologies, Foster City, CA, USA) and stored at −80 °C until RNA isolation.

### Histopathological evaluation

Paraffin-embedded samples were processed and stained as previously described^29^. To determine the severity of interstitial fibrosis and chronic inflammation, a board-certified veterinary anatomic pathologist (REC) evaluated 5 or 20 randomly chosen 20x fields of renal cortex based on core size for each biopsy. For interstitial fibrosis, a score of 0 to 3 was assigned for each field based on the degree of tubulointerstitial architecture distortion caused by fibrosis: 0 - no fibrosis, 1 - fibrosis present but no distortion, 2 – moderate distortion, and 3 – severe distortion. For chronic inflammation, 0 – no inflammatory cells, 1 – scattered inflammatory cells, 2 – aggregates of inflammatory cells that separate or replace tubules, and 3 – diffusely distributed inflammatory cells. Statistical analysis comparing the average scores between groups was performed using bootstrap in R (version 3.2.4) to construct simultaneous 95% confidence intervals for all 3 pairwise comparisons of mean fibrosis and chronic inflammation scores at each of the latter 2 disease stages.

### RNA isolation and sequencing

The MirVana miRNA Isolation Kit (Ambion, Austin, TX, USA) was used to isolate total RNA from homogenized kidney tissue according to the manufacturer’s instructions. The library preparation, sequencing, and initial quality check were performed by the Texas A&M AgriLife Genomics and Bioinformatics Service (http://www.txgen.tamu.edu/). RNA integrity was assessed by the Agilent 2100 Bioanalyzer (Agilent Technologies, Santa Clara, CA, USA). The average RIN was 3.4, and the average RNA yield was 86.2 ng/μL (Supplementary Table S3). To compensate for the low-input samples, we use the TruSeq Stranded Total RNA Library Prep Kit with Ribo-zero Gold (Illumina, San Diego, CA, USA), based on the best practice for RNA with variable qualities^21^ (to remove both cytoplasmic and mitochondrial rRNA) and its compatibility with canine samples. Samples were then sequenced using the Illumina Genome Analyzer (HiSeq. 2500v4 High Output). Raw sequencing data were submitted to the NCBI sequence read archive (SRA) (Accession: SRP101707; Samples: SAMN06560417, SAMN06560429-51; BioProject: PRJNA378728).

### Data analysis

FastQC (version 0.11.2) was used for quality control to ensure that the quality value was above Q30. The canine (*Canis lupus familiaris*) genome FASTA file (ftp://ftp.ensembl.org/pub/current_fasta/canis_familiaris/dna/Canis_familiaris.CanFam3.1.dna.toplevel.fa.gz) and gene annotation GTF file (CanFam 3.1 assembly; ftp://ftp.ensembl.org/pub/current_gtf/canis_familiaris/) were obtained from Ensembl. Although RNA-seq is a popular research tool, there is no gold standard for analyzing RNA-seq data. Among the available tools, we chose up-to-date open source tools for mapping, retrieving read counts, and differential analysis. We used HISAT2^22^ (version 2.0.3-beta) to generate indexes and to map reads to the canine genome. For assembly, we chose SAMtools (version 1.2) and the “union” mode of HTSeq^23^ (version 0.6.1), as the gene-level read counts could provide more flexibility in the differential expression analysis. Both HISAT2 and HTSeq analyses were conducted using the high performance research computing resources provided by Texas A&M University (http://hprc.tamu.edu) in the Linux operating system (version 2.6.32). Differential expression and statistical analysis were performed using DESeq2 (release 3.3) in R (version 3.2.4). DESeq2^24^ was chosen as it is a popular parametric tool that provides a descriptive and continually updated user manual (https://bioconductor.org/packages/release/bioc/vignettes/DESeq2/inst/doc/DESeq2.html). DESeq2 internally corrects for library size, so it is important to provide un-normalized raw read counts as input. We used variance stabilizing transformation to account for differences in sequencing depth. P-values were adjusted for multiple testing using the Benjamini-Hochberg procedure^60^. A false discovery rate adjusted p-value (i.e., q-value) < 0.05 was set for the selection of DE genes.

### Gene ontology (GO), pathway, and upstream regulator analysis

Gene ontology and PANTHER pathway analyses were performed with the PANTHER Overrepresentation Test (released on July 15, 2016) in PANTHER^25^ version 11.1 (http://www.pantherdb.org/, released on October 24, 2016). This program supports the canine genome. Also, QIAGEN’s Ingenuity Pathway Analysis (IPA, QIAGEN Redwood City, www.qiagen.com/ingenuity) was used to provide overrepresented orthologous genes in human, mouse, and rat databases and to identify orthologous pathways and upstream regulators in our data. PANTHER used the binomial test and Bonferroni correction for multiple testing, while IPA used the right-tailed Fisher Exact test and displayed z-scores to indicate whether a potential regulator was activated or inhibited. We used the default settings for statistical analysis in both the PANTHER pathway and IPA. In PANTHER, only pathways and GO terms with fold enrichment > 0.2 were listed. In IPA, p-value < 0.05 and fold change > 2 were set as cutoff values.

### Immunohistochemistry (IHC)

Three-micrometer, formalin-fixed, paraffin-embedded renal cortex sections of both affected and control dogs were stained for CD3 (n = 24) and CD20 (n = 9). After deparaffinization, the sections were placed in citrate buffer (pH 6.0) for antigen retrieval, using a pressure cooker (Decloaking Chamber, Biocare Medical). Endogenous peroxidase activity and non-specific protein binding were blocked with 3% hydrogen peroxide and Sniper protein block (Biocare Medical), respectively. After blocking, the sections were incubated with primary antibodies CD3 (1:300 dilution; Dakocytomation, Carpinteria, CA) and CD20 (1:500 dilution; Thermo Scientific, Fremont, CA) for 1 hour at room temperature, then incubated with MACH 2 polymer for 30 minutes at room temperature. DAB (Vector Laboratories, Burlingame, CA) was used as the chromogen to demonstrate sites of antibody-antigen reaction. Mayer’s hematoxylin was used for the counterstain. Photographs were obtained using a SPOT Insight 2Mp FW Color Mosaic Camera (Diagnostic Instruments, Inc., Sterling Heights, MI) and the SPOT software (version 5.2).

### Data Availability

Raw sequencing reads generated from this study are deposited at the NCBI sequence read archive (SRA) under accession SRP101707 (Samples: SAMN06560417, SAMN06560429-51; BioProject: PRJNA378728).

## Electronic supplementary material

Supplementary File S1

Supplementary Table S4

Supplementary Table S5

Supplementary Table S6

Supplementary Table S7
