# Author Correction: RNA-seq of serial kidney biopsies obtained during progression of chronic kidney disease from dogs with X-linked hereditary nephropathy

**DOI:** 10.1038/s41598-020-59299-3

**Published:** 2020-02-06

**Authors:** Candice P. Chu, Jessica A. Hokamp, Rachel E. Cianciolo, Alan R. Dabney, Candice Brinkmeyer-Langford, George E. Lees, Mary B. Nabity

**Affiliations:** 1 Department of Veterinary Pathobiology, College of Veterinary Medicine and Biomedical Sciences, Texas A&M University, College Station, TX USA; 2 Department of Veterinary Biosciences, College of Veterinary Medicine, The Ohio State University, Columbus, OH USA; 3 Department of Statistics, College of Science, Texas A&M University, College Station, TX USA; 4 Department of Veterinary Integrative Biomedical Sciences, College of Veterinary Medicine and Biomedical Sciences, Texas A&M University, College Station, TX USA; 5 Department of Small Animal Clinical Sciences, College of Veterinary Medicine and Biomedical Sciences, Texas A&M University, College Station, TX USA

Correction to: *Scientific Reports* 10.1038/s41598-017-16603-y, published online 01 December 2017

The Acknowledgements section in this Article is incorrect.

“This work was funded by the National Institutes of Health (DK57676 and DK64273) and the Texas A&M Genomics Seed Grant Program. This study was presented in part at the 2016 American College of Veterinary Pathologists/American Society for Veterinary Clinical Pathology Annual Meeting in New Orleans, LA, December 2016. We would like to thank Dr. Ivan Ivanov for offering valuable suggestions for this study and providing RNA-seq analysis training through the Center for Translational Environmental Health Research (http://www.ctehr.org), Dr. Michael Dicken and all the personnel in the TAMU High Performance Research Computing (http://hprc.tamu.edu) for providing useful resources and instant help during data analysis, Dr. Michael Love for his rapid response in the Bioconductor online forum (https://support.bioconductor.org/), Dr. Andy Ambrus for the immunohistochemistry staining, Mia Aguilar and Mary Sanders for their technical assistance with RNA isolation and sample collection from dogs, respectively, and Cheng-An Richard Chang for guiding us through the first step of RNA-seq analysis. Special thanks to Dr. Barbara Gastel and Leah Poffenberger for their editing assistance on this manuscript.”

should read:

“This work was funded by the National Institutes of Health (DK57676 and DK64273) and the Texas A&M Genomics Seed Grant Program. This study was presented in part at the 2016 American College of Veterinary Pathologists/American Society for Veterinary Clinical Pathology Annual Meeting in New Orleans, LA, December 2016. We would like to thank Dr. Ivan Ivanov for offering valuable suggestions for this study and providing RNA-seq analysis training through the Center for Translational Environmental Health Research (http://www.ctehr.org), Dr. Michael Dickens and all the personnel in the TAMU High Performance Research Computing (http://hprc.tamu.edu) for providing useful resources and instant help during data analysis, Dr. Michael Love for his rapid response in the Bioconductor online forum (https://support.bioconductor.org/), Dr. Andy Ambrus for the immunohistochemistry staining, Mia Aguilar and Mary Sanders for their technical assistance with RNA isolation and sample collection from dogs, respectively, and Cheng-An Richard Chang for guiding us through the first step of RNA-seq analysis. Special thanks to Dr. Barbara Gastel and Leah Poffenberger for their editing assistance on this manuscript. The open access publishing fees for this article have been covered by the Texas A&M University Open Access to Knowledge Fund (OAKFund), supported by the University Libraries and the Office of the Vice President for Research.”

