# Supplementary material for: RNA-seq of serial kidney biopsies obtained during progression of chronic kidney disease from dogs with X-linked hereditary nephropathy
Source: Sci Rep. 2017 Dec 1;7:16776. doi: 10.1038/s41598-017-16603-y (PMC5711945; doi:10.1038/s41598-017-16603-y)
Supplement: Supplementary file 1 — Supplementary File S1 [file 41598_2017_16603_MOESM1_ESM.doc]

**RNA-Seq of Serial Kidney Biopsies Obtained During Progression of Chronic Kidney Disease from Dogs with X-Linked Hereditary Nephropathy**

Candice P. CHU, Jessica A. HOKAMP, Rachel E. CIANCIOLO, Alan R. DABNEY, Candice L. BRINKMEYER-LANGFORD, George E. LEES, Mary B. NABITY

**Supplementary File S1**

**Supplementary Table S1. Mean fibrosis scores and confidence intervals.**

|  | mean fibrosis score (0-3) in different groups | | |  |
| --- | --- | --- | --- | --- |
| time points | rapid group | slow group | control group | confidence Interval of difference in mean fibrosis score (*statistically significant) |
| T2 | - | 0.4 | 0 | 0.20 0.60* |
| T2 | 1.4 | - | 0 | 0.40 2.70* |
| T2 | 1.4 | 0.4 | - | -0.13 2.36 |
| T3 | - | 1.3 | 0 | 0.90 1.60* |
| T3 | 2.7 | - | 0 | 2.10 3.00* |
| T3 | 2.7 | 1.3 | - | 0.75 2.00* |


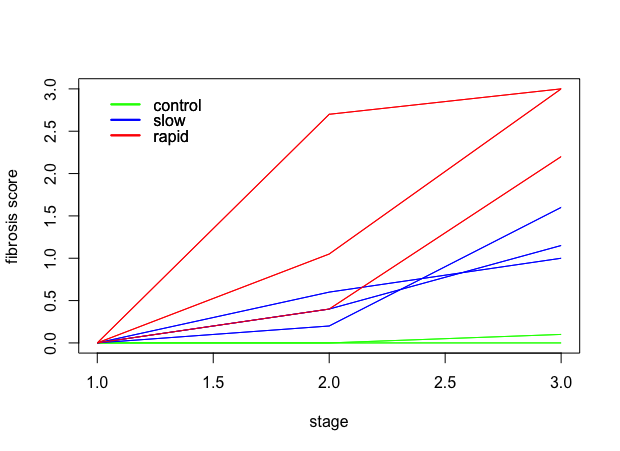


**Supplementary Figure S1. Trends of changes in fibrosis scores for individual dogs over time.**

**Supplementary Table S2. Mean chronic inflammation scores and confidence intervals.**

|  | mean chronic inflammation score (0-3) in different groups | | |  |
| --- | --- | --- | --- | --- |
| time points | rapid group | slow group | control group | confidence Interval of difference in mean chronic inflammation score (*statistically significant) |
| T2 | - | 0.67 | 0 | 0.40 0.80* |
| T2 | 1.12 | - | 0 | 0.20 2.20* |
| T2 | 1.12 | 0.67 | - | -0.60 1.66 |
| T3 | - | 1.18 | 0 | 1.00 1.55* |
| T3 | 1.78 | - | 0 | 0.80 2.55* |
| T3 | 1.78 | 1.18 | - | -0.56 1.55 |


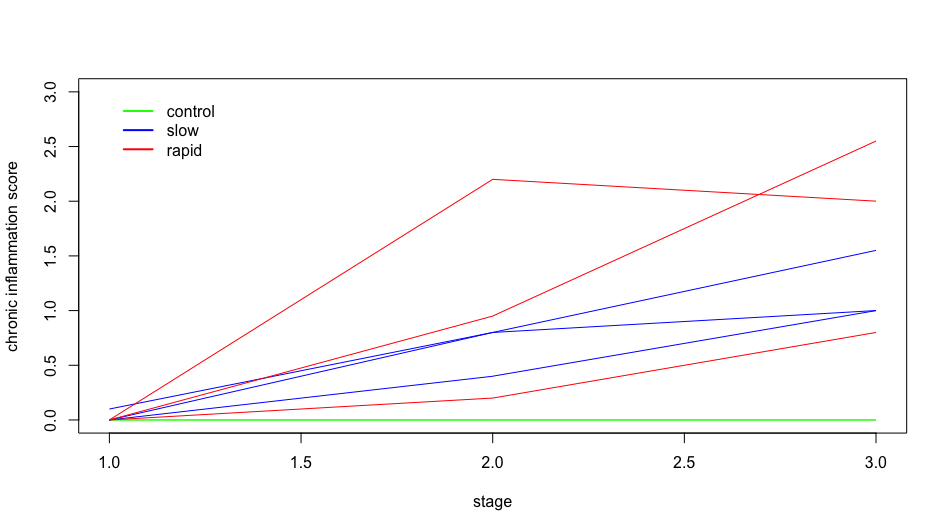


**Supplementary Figure S2. Trends of changes in chronic inflammation scores for individual dogs over time.**

**Supplementary Table S3**. Overview of RNA-Seq Mapping.

|  | Dog | Input Paired-end Reads | Overall Alignment rate  (%) | Uniquely mapped Paired-end reads  (%) | Unmapped reads  (%) | Compressed File size  (GB) | Number of genes in raw counts | RNA Concentration  (ng/ul) | RIN |
| --- | --- | --- | --- | --- | --- | --- | --- | --- | --- |
| T1 | Rapid_1 | 29514770 | 95.55 | 77.05 | 4.45 | 4.6 | 19760 | 14.85 | 1.6 |
|  | Rapid_2 | 33881080 | 95.14 | 74.24 | 4.86 | 5.2 | 19997 | 197.82 | 3.8 |
|  | Rapid_3 | 25344592 | 95.97 | 74.58 | 4.03 | 4 | 19333 | 123.64 | 2 |
|  | Slow_1 | 27891909 | 95.83 | 75.34 | 4.17 | 4.2 | 19772 | 98.12 | 2 |
|  | Slow_2 | 29895251 | 95.53 | 74.71 | 4.47 | 4.5 | 19919 | 48.41 | 2 |
|  | Slow_3 | 31434595 | 95.6 | 78.04 | 4.4 | 4.9 | 19912 | 109.11 | 3.3 |
|  | Control_1 | 34783623 | 91.2 | 73.92 | 8.8 | 5.2 | 20343 | 33.64 | 2.6 |
|  | Control_2 | 34279256 | 94.23 | 69.91 | 5.77 | 5.4 | 19797 | 59.05 | 2.7 |
| T2 | Rapid_1 | 28435157 | 94.66 | 75.96 | 5.34 | 4.5 | 20436 | 51.04 | 5.3 |
|  | Rapid_2 | 30077789 | 94.52 | 71.63 | 5.48 | 4.7 | 18924 | 38.45 | 6.8 |
|  | Rapid_3 | 26349776 | 95.22 | 75.23 | 4.78 | 4.1 | 20415 | 19.63 | 1.6 |
|  | Slow_1 | 34079092 | 95.69 | 73.85 | 4.31 | 5.2 | 19685 | 213.87 | 2.8 |
|  | Slow_2 | 29270490 | 95.43 | 74.73 | 4.57 | 4.5 | 20154 | 20.45 | 3.1 |
|  | Slow_3 | 30077556 | 95.45 | 78.08 | 4.55 | 4.8 | 20321 | 50.93 | 4.3 |
|  | Control_1 | 38146587 | 95.5 | 73.2 | 4.5 | 5.8 | 19549 | 165.58 | 4.2 |
|  | Control_2 | 35945644 | 95.39 | 72.64 | 4.61 | 5.5 | 20124 | 35.15 | 1 |
| T3 | Rapid_1 | 33628572 | 94.33 | 75.21 | 5.67 | 5.4 | 21026 | 132.45 | 4.4 |
|  | Rapid_2 | 28400443 | 94.58 | 73.77 | 5.42 | 4.5 | 20281 | 123.43 | 3.6 |
|  | Rapid_3 | 27557955 | 94.12 | 74.01 | 5.88 | 4.4 | 21319 | 40.88 | 1.1 |
|  | Slow_1 | 30104374 | 94.35 | 71.38 | 5.65 | 4.7 | 19766 | 93.99 | 6 |
|  | Slow_2 | 29966690 | 94.19 | 72.14 | 5.81 | 4.7 | 20816 | 126.33 | 4 |
|  | Slow_3 | 32095615 | 95.05 | 77.31 | 4.95 | 5.2 | 21148 | 22.52 | 2.9 |
|  | Control_1 | 35069833 | 95.68 | 74.76 | 4.32 | 5.2 | 19863 | 18.73 | 4.1 |
|  | Control_2 | 37638711 | 95.53 | 73.27 | 4.47 | 5.7 | 19519 | 231.17 | 6 |


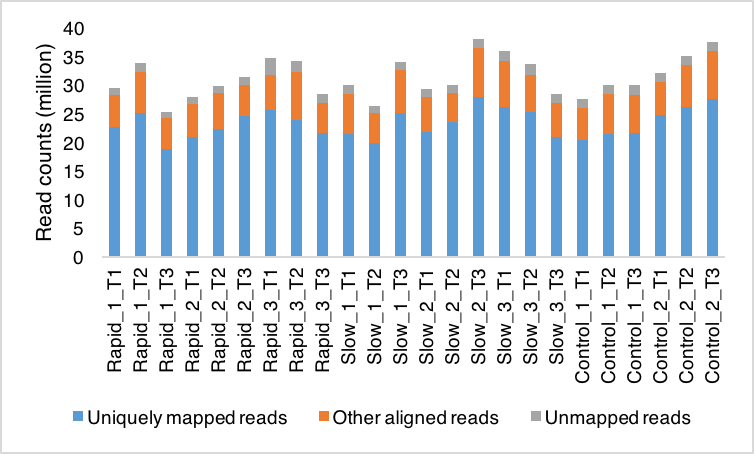


**Supplementary Figure S3. Overview of RNA-Seq Mapping.**


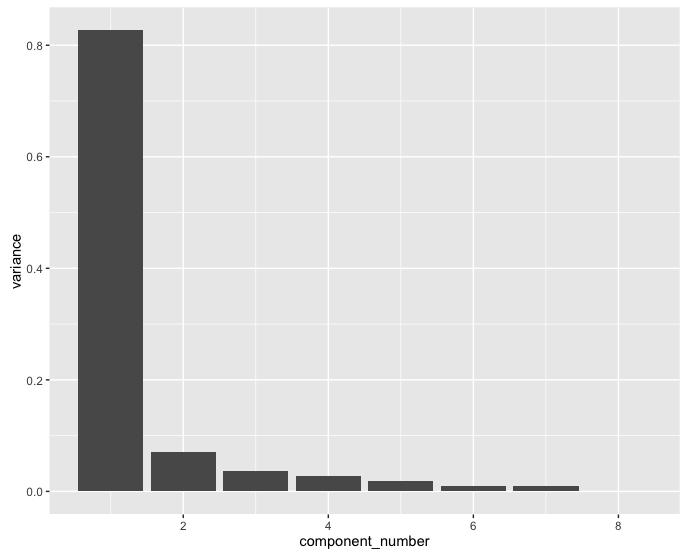


**Supplementary Figure S4-1. PCA scree plot at T1.**


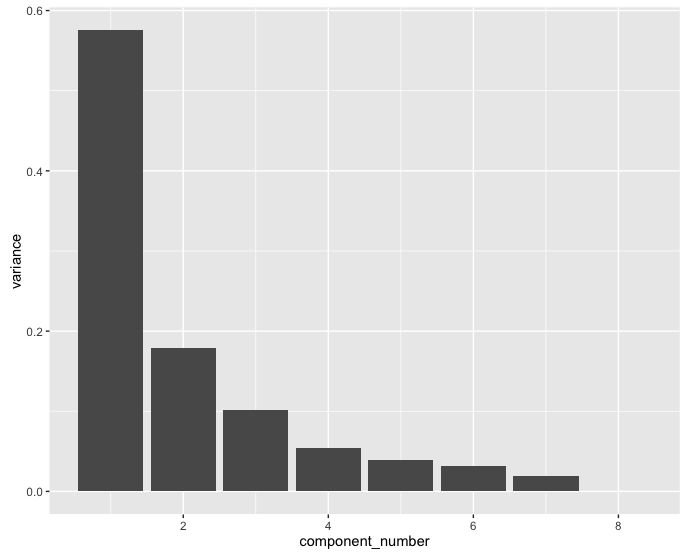


**Supplementary Figure S4-2. PCA scree plot at T2.**


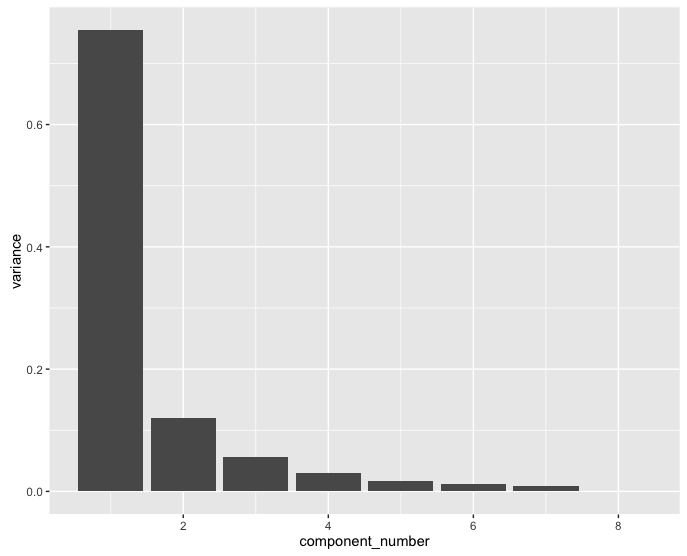


**Supplementary Figure S4-3. PCA scree plot at T3.**
